# Declared impact of the US President’s statements and campaign statements on Latino populations’ perceptions of safety and emergency care access

**DOI:** 10.1371/journal.pone.0222837

**Published:** 2019-10-30

**Authors:** Robert M. Rodriguez, Jesus R. Torres, Jennifer Sun, Harrison Alter, Carolina Ornelas, Mayra Cruz, Leah Fraimow-Wong, Alexis Aleman, Luis M. Lovato, Angela Wong, Breena Taira

**Affiliations:** 1 Department of Emergency Medicine, University of California San Francisco, San Francisco, California, United States of America; 2 Highland Hospital-Alameda Health System, Oakland, California, United States of America; 3 Olive View UCLA Medical Center–University of California Los Angeles School of Medicine, Los Angeles, California, United States of America; University of Washington School of Medicine, UNITED STATES

## Abstract

Statements about building walls, deportation and denying services to undocumented immigrants made during President Trump’s presidential campaign and presidency may induce fear in Latino populations and create barriers to their health care access. To assess how these statements relate to undocumented Latino immigrants’ (UDLI) and Latino legal residents/citizens’ (LLRC) perceptions of safety and their presentations for emergency care, we conducted surveys of adult patients at three county emergency departments (EDs) in California from June 2017 to December 2018. Of 1,684 patients approached, 1,337 (79.4%) agreed to participate: 34.3% UDLI, 36.9% LLRC, and 29.8% non-Latino legal residents/citizens (NLRC). The vast majority of UDLI (95%), LLRC (94%) and NLRC (85%) had heard statements about immigrants. Most UDLI (89%), LLRC (88%) and NLRC (87%) either thought that these measures were being enacted now or will be enacted in the future. Most UDLI and half of LLRC reported that these statements made them feel unsafe living in the US, 75% (95% CI 70–80%) and 51% (95% CI 47–56%), respectively. More UDLI reported that these statements made them afraid to come to the ED (24%, 95% CI 20–28%) vs LLRC (4.4%, 95% CI 3–7%) and NLRC (3.5%, 95% CI 2–6%); 55% of UDLI with this fear stated it caused them to delay coming to the ED (median delay 2–3 days). The vast majority of patients in our California EDs have heard statements during the 2016 presidential campaign or from President Trump about measures against undocumented immigrants, which have induced worry and safety concerns in both UDLI and LLRC patients. Exposure to these statements was also associated with fear of accessing emergency care in some UDLIs. Given California’s sanctuary state status, these safety concerns and ED access fears may be greater in a nationwide population of Latinos.

## Introduction

The Pew Research Center estimates that approximately 8 million undocumented Latino immigrants (UDLI) lived in the United States in 2016 [1]. Given the lack of availability of other health care options including primary care, emergency departments (EDs) serve as the primary health care access point and safety net for UDLI [2–4]. Even though unfettered access to emergency care—regardless of citizenship or ability to pay—is protected by law under the Emergency Medical Treatment and Active Labor Act (EMTALA) of 1986 [5], UDLI face a number of obstacles when coming to EDs. In a 2013 study, we demonstrated that fear of discovery and deportation deters some UDLI from presenting for emergency care [6]. Since that time, US policy toward immigrants has evolved considerably, and immigration has become a heated topic across the US. Most notably, President Trump made deportation of “illegal” immigrants—particularly those from Latin America—a cornerstone of his 2016 campaign [7–9], and he has continued to promote a hardline stance toward immigrants throughout his presidency [10–12]. Although the effects of the president’s statements and policy shift on attitudes of US citizens and voters have been well documented by traditional news media surveys and polling organizations [13,14], very little is known about the impact on immigrants themselves. Threats of deportation may induce safety concerns, anxiety and other mental health problems in UDLI. Statements about denying health care (and other) services may generate misconceptions that increase their fear of emergency care access and thereby compromise the public health and safety net function of EDs.

Beyond the possible impact on UDLI, the president’s statements about immigrants may also affect the 58 million Latino legal residents/citizens (LLRC) of the US, many of whom have undocumented immigrant family and friends. Almost 6 million US citizen children live with an undocumented family member [15]. With these issues in mind, we conducted this multicenter survey study to examine whether statements about immigrants made during the 2016 presidential campaign or by President Trump affected Californian UDLI’s and LLRC’s perceptions of safety and fear of accessing emergency care. Specifically, we sought to determine 1) the proportion of UDLI and LLRC who had heard statements about deportation and denying services to undocumented immigrants from the president or during the campaign; 2) whether these statements affected their feelings of safety living in the US; and 3) whether these statements made them afraid to come to the ED for care.

## Methods

### Ethics statement

We obtained institutional review board (IRB) approval from the University of California of San Francisco Committee on Human Research, the Olive-View UCLA Medical Center IRB and the Highland Hospital—Alameda Health System IRB to conduct this survey study. Because the surveys were anonymous with no identifying information, a signed consent would unnecessarily add identifying information and add risk of discovery for these UDLI. The IRBs therefore recommended scripted, verbal consent. We recorded whether patients agreed to participate as a yes/no field at the initiation of the surveys.

### Study design and setting

From mid-June 2017 to mid-December 2018, we conducted this survey study at three urban county hospitals in California (Zuckerberg San Francisco General Hospital [San Francisco, CA], Olive View-UCLA Medical Center [Sylmar, CA] and Highland Hospital—Alameda Health System [Oakland, CA]) with annual ED censuses of 77,000, 60,000 and 73,000 patients, respectively. At these EDs, 45.3% of visits were by patients of self-declared Latino ethnicity in 2017. We obtained IRB approval from each institution to conduct this anonymous survey study with scripted, verbal consent.

### Survey development

After consultation with health literacy experts with expertise in survey design, we adapted our 2013 survey instrument to include questions assessing the following primary outcome variables: 1) whether respondents had heard statements about measures against undocumented immigrants during the presidential campaign or from President Trump; 2) whether these statements had affected their feelings of safety living in the US; and 3) whether these statements had induced fear in accessing emergency care. We pilot tested the final instrument, consisting of yes/no, multiple choice, free text response, and numerical analog questions, on ten UDLI and LLRC to ensure brevity and comprehension of questions with consistent responses. We found excellent understanding and response consistency and that the survey took less than eight minutes on average to administer (S1 Fig and S2 Fig).

### Participants and enrollment

Using a convenience sampling method consisting of enrollment when study personnel were available (typically four- to six-hour time blocks on weekdays), we screened ED tracking boards for eligible adult patients. We excluded patients with any of the following characteristics: 1) trauma; 2) transfer from another facility; 3) inability to participate in an interview because of intoxication, altered mental status or critical illness; 4) incarceration; and 5) on psychiatric hold. In order to enroll similar numbers of our three groups (UDLI, LLRC and non-Latino legal residents/citizens [NLRC]), we examined our central database tally of the participant group assignments monthly. When the total number of participants in one group fell to 20% lower than the other groups, we shifted toward approaching more patients in that group. We excluded patients after interviews if they were vacationing or were non-Latino undocumented immigrants.

### Survey administration

Physicians, students and other research personnel, most of whom were fluent Spanish speakers, received orientation sessions and shadowed the principal investigators during initial surveys to ensure standard survey technique. After scripted verbal consent, these study personnel read survey questions to participants directly from data collection forms in their preferred language (fluent Spanish speakers only for Spanish-speaking participants) in private ED areas. Without recording any numbers or identifying information, we reviewed ED registration records to determine whether respondents had a social security number (SS#). After the surveys, interviewers assured subjects that EDs maintain confidentiality regarding immigration status and provided information sheets about local resources for primary medical care and social services support.

### Data management and analysis

Using double checks on entry, we entered data into REDCap hosted by the University of California, San Francisco [16]. We used STATA v 15.1 (StataCorp, College Station, TX) for analyses, summarizing patient characteristics as raw counts and frequency percent and key survey questions as percentages (proportions) with 95% confidence intervals (CIs). Non-responses to individual questions were not included in the denominators of these proportions.

We categorized participants after the surveys into UDLI, LLRC, and NLRC groups by the following direct questions: *Do you identify as being of Latino origin*? *Are you a legal resident/citizen of the United States*? Our three primary outcomes were 1) the proportions of UDLI, LLRC and NLRC who had heard statements about measures against immigrants from the president or during the campaign; 2) whether these statements made them worry or feel unsafe living in the US; and 3) whether these statements made them afraid to come to the ED for care.

To assess differences between groups, we compared 95% CIs around differences in proportions. We calculated odds ratios (ORs) to describe the association of patient characteristics (age, sex, identification as Latino, legal US resident status, having health insurance, having a doctor/clinic for regular care, having housing, and belief that the statements about immigrants are being/will be enacted) with the primary outcomes of feeling unsafe in the US and being afraid to come to the ED. Additionally, we estimated adjusted odds ratios (aORs) of characteristics that showed OR significance, while adjusting for interactions, using logistic regression to examine which factors continued to demonstrate independent association with these two outcomes. To have no more than a 5% CI around the point estimates for prevalence of safety concerns and fear of coming to the ED, we calculated a priori that we would need to enroll at least 384 patients in each group.

We conducted two secondary analyses of the two primary outcomes of feeling unsafe in the US and being afraid to come to the ED on the UDLI sub-group of patients. We compared 1) recent immigrants (< 5 years) versus non-recent immigrants (> 5 years), and 2) immigrants surveyed in the first 12 months (late June 2017 –June 2018) versus the last six months (July–December 2018) of the study time period.

## Results

Of 1,684 patients approached, 1,337 (79.4%) agreed to participate. We excluded 16 patients because they were non-Latino undocumented immigrants and three because they were just visiting the US. Of the remaining 1,318 patients, 452 (34.3%) were UDLI, 473 (35.9%) LLRC, and 393 (29.8%) NLRC. Enrollment and categorization of participants at the three sites was (501: 34% UDLI, 38% LLRC and 28% NLRC); (418: 36% UDLI, 37% LLRC and 27% NLRC); and (399: 33% UDLI, 32% LLRC and 35% NLRC).

On review of ED registration records, 15% of UDLI, 89% of LLRC and 93% of NLRC had SS#s. Compared to LLRC and NLRC, UDLI were younger, more often female, less likely to have health insurance, and less likely to have primary care. Most UDLI (93%) had seen a doctor in the US previously. Spanish was the primary language of 97% of UDLI with 78% of them reporting little to no English proficiency; 22% stated that they were brought to the US as children. UDLI without primary care were more likely to get their usual medical care in an ED (Table 1).

**Table 1:** Patient characteristics sorted according to study groups.

	UDLI	LLRC	NLRC
	n (%)	n (%)	n (%)
**Total Number**	452 (34)	473 (36)	393 (30)
**Male**	200 (44)	260 (55)	221 (56)
**Median age in years (IQR)**	40 (32, 51)	45 (28, 58)	48 (33, 60)
Primary Language*			
English	17 (4)	201 (42)	349 (89)
Spanish	437 (97)	308 (65)	3 (1)
Other	6 (1)	7 (1)	60 (15)
**English Proficiency**			
Not at all	104 (23)	27 (6)	2 (0.5)
A little	247 (55)	117 (25)	10 (3)
Most of it	68 (15)	81 (17)	33 (8)
Completely	38 (8)	249 (53)	349 (89)
**Have Health Insurance**	315 (70)	384 (81)	332 (84)
Private	15 (3)	41 (9)	43 (11)
Medicare/Medi-Cal	177 (39)	328 (69)	285 (73)
Healthy SF/My Health LA/HealthPAC**	133 (29)	39 (8)	17 (4)
**Have Housing**	437 (97)	448 (95)	346 (88)
**Have PCP**	271 (60)	312 (66)	253 (64)
**If no PCP, where do you get care?**			
Clinic	24 (17)	41 (30)	33 (31)
Emergency Department	117 (83)	96 (70)	75 (69)

PCP–primary care provider; IQR–interquartile range; UDLI–Undocumented Latino Immigrant; LLRC–Latino legal resident/citizen; NLRC–Non-Latino resident/citizen.

*Patients could declare more than one primary language

**Healthy SF, My Health LA and HealthPAC are Department of Public Health programs in San Francisco, Los Angeles and Alameda counties that provide free or affordable health services to those who are not eligible for public insurance (Medi-Cal and Medicare) and are below a certain income threshold.

**Fig 1:**
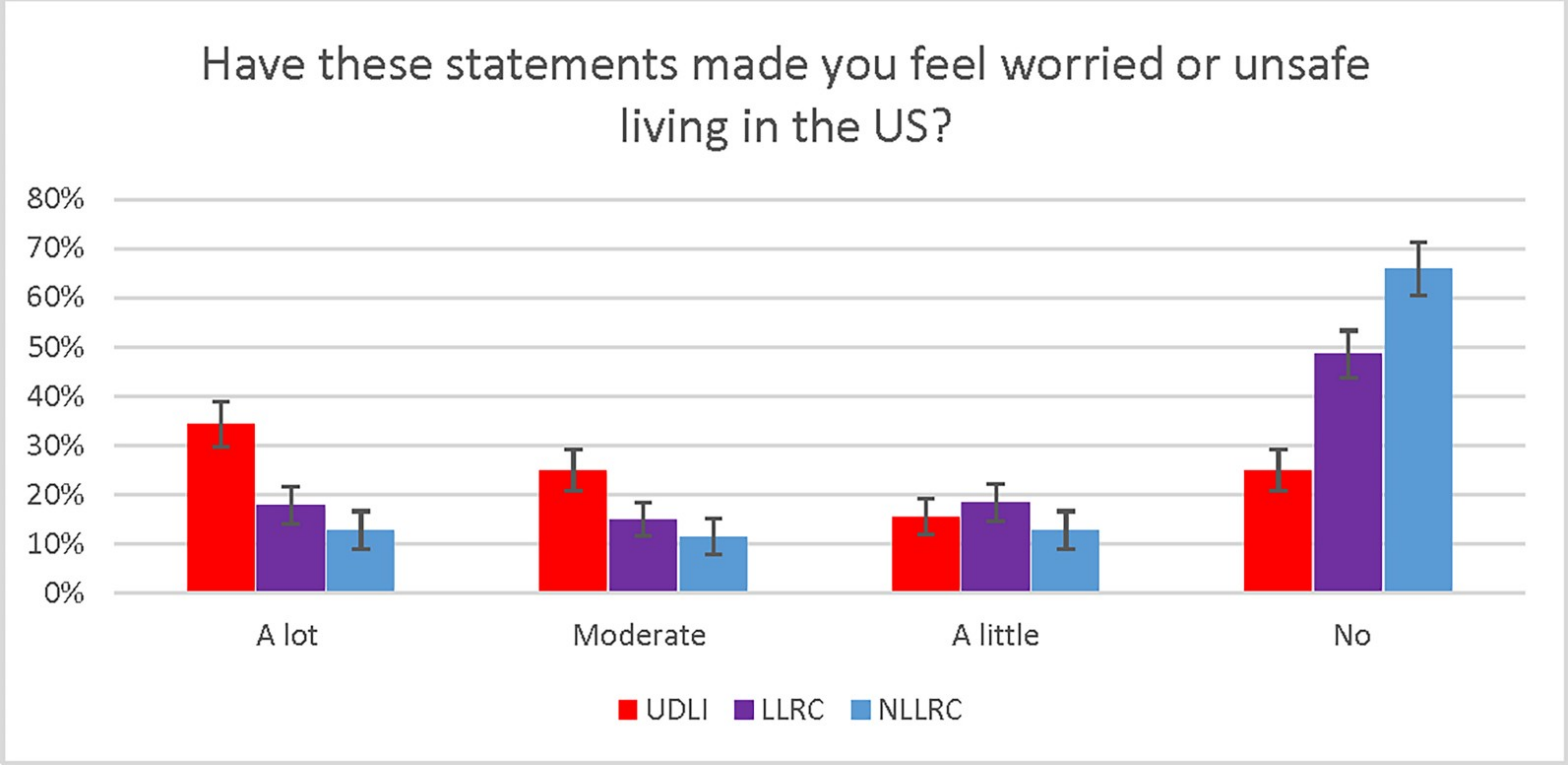
Perceived impact of the president’s statements about immigrants on feelings of safety. UDLI–Undocumented Latino immigrant; LLRC–Latino legal resident/citizen; NLRC–Non-Latino resident/citizen; US–United States. Error bars represent 95% confidence intervals.

Nearly all UDLI (99.3%), LLRC (99.8%), and NLRC (97.4%) knew the name of the US president (President Trump). Greater percentages of UDLI (95%, 95% CI 92–96%) and LLRC (94%, 95% CI 91–96%) had heard statements about measures against immigrants than NLRC (85%, 95% CI 81–88%). High percentages of UDLI respondents had heard that President Trump wants to deport immigrants (92%), deny immigrants services (87%) and prevent immigrants from getting health care in the US (79%). Of those who had heard these statements, most UDLI (89%), LLRC (88%) and NLRC (87%) thought that these measures were being enacted now or will be enacted in the future. When asked whether these statements made them feel worried or unsafe living in the US, most UDLI (75%; 95% CI 71–79%) and approximately half of LLRC (51%; 95% CI 47–56%) responded Yes “a little”, “somewhat”, or “a lot” (Table 2 and Fig 1).

**Table 2:** Responses to key questions sorted according to study groups.

	UDLI	LLRC	NLRC
	% (95% CI)	% (95% CI)	% (95% CI)
**Believe that providers treat US citizens different than non-US citizens?**			
Yes	11 (8–14)	11 (8–14)	12 (9–16)
No	81 (77–85)	75 (7–78)	60 (55–65)
Unsure	8 (6–11)	15 (12–18)	30 (26–35)
**Believe that hospital staff report illegal immigrants to the authorities?**			
Yes	4 (2–6)	4 (3–7)	7 (5–10)
No	81 (77–84)	76 (71–79)	60 (55–65)
Unsure	15 (12–19)	20 (17–24)	33 (28–38)
**Did anyone ask about your citizenship?**			
Yes	5 (3–8)	7 (5–10)	6 (4–9)
No	93 (90–95)	81 (77–84)	89 (85–92)
Unsure	2 (1–4)	2 (1–4)	6 (4–9)
**Do you know someone (friends or family) that did not come to the ED out of fear of being discovered as undocumented?**			
Yes	24 (21–28)	26 (22–30)	15 (11–19)
**Have you heard any of these statements? The president wants to:**			
Build a wall	92 (89–95)	92 (89–94)	77 (73–81)
Deport immigrants	92 (89–95)	91 (88–94)	77 (72–81)
Deny services	87 (83–90)	80 (76–83)	63 (58–68)
Prevent immigrant from working	86 (83–89)	81 (77–85)	59 (53–63)
Prevent immigrants from getting health care	79 (74–82)	73 (68–76)	53 (48–58)
**Do you believe any of these are currently occurring or will happen?**			
No, not now and will never happen	11 (8–14)	12 (10–16)	13 (1–17)
Not right now, but will happen in the future	31 (26–35)	29 (25–33)	19 (16–24)
Some are occurring now	43 (38–48)	41 (37–46)	33 (28–38)
All are occurring now	10 (7–13)	12 (10–16)	8 (6–11)
**Have these statements made you feel worried or unsafe living in the US?**			
No, they have not affected me at all	25 (21–30)	49 (44–53)	66 (60–71)
Yes, little worried or unsafe	16 (12–20)	18 (15–22)	13 (9–17)
Yes, somewhat (moderate) worried or unsafe	25 (21–30)	15 (12–19)	12 (8–16)
Yes, they've affected me a lot	34 (30–40)	18 (14–19)	13 (9–17)
**Did any of these statements make you afraid to come to the ED?**			
No, not at all	76 (72–81)	96 (93–97)	96 (94–98)
Yes, a little	11 (8–14)	2 (1–4)	1 (0–3)
Yes, a moderate amount	8 (6–11)	2 (1–3)	1 (0–3)
Yes, a lot	5 (3–7)	1 (0–2)	1 (0–3)

UDLI–Undocumented Latino immigrant; LLRC–Latino legal resident/citizen; NLRC–Non-Latino resident/citizen; CI–confidence interval

When asked whether these statements about immigrants made them afraid to come to the ED, a greater percentage of UDLI as compared to LLRC and NLRC reported Yes “a little”, “a moderate amount”, or “a lot” (UDLI 24%, 95% CI 20–28%; LLRC 4%, 95% CI 3–7%; and NLRC 4%, 95% CI 2–6%). Of the UDLI who had this fear, 55% stated that they delayed coming to the ED (median delay of 2–3 days). Similar percentages of UDLI (24%, 95% CI 21–28%) and LLRC (26%, 95% CI 22–30%) reported that they had friends or family members who had not come to the ED because of fear of discovery, greater than the 15% (95% CI 11–19%) of NLRC who reported having friends/family who had not come to the ED (Table 2).

Only undocumented status (aOR 2.8; 95% CI 2.1–3.9), belief that the president’s immigrant statements are being/will be enacted (aOR 2.4; 95% CI 1.6–3.5), and identification as Latino (aOR 1.5; 95% CI 1.1–2.1) were independently associated with feeling unsafe in the US. Only undocumented status (aOR 7.4; 95% CI 4.4–13.3) and belief that the president’s immigrant statements are being/will be enacted (aOR 2.1; 95% CI 1.0 to 4.8) were independently associated with fear of coming to the ED (Table 3).

**Table 3:** Association of characteristics with primary outcomes of feeling unsafe in the US and feeling afraid to come to the ED because of the president’s anti-immigrant statements: ORs and aORs.

Characteristic	Feel unsafe living in US because of statements OR (95% CI)	Afraid to come to ED because of statements OR (95% CI)
Age (unit = 10 years)	0.9 (0.8–0.9)*	0.9 (0.8–1.1)
Female	2.3 (1.8–2.9)*	1.2 (0.8–1.7)
Identify as Latino	2.6 (2.0–3.5)*	4.7 (2.5–10.2)*
Non-insured	1.1 (0.8–1.5)	1.4 (0.9–2.2)
No regular clinic or doctor for care	0.9 (0.7–1.2)	1.4 (0.9–2.0)
Not housed	0.6 (0.4–1.1)	1.5 (0.7–2.9)
Not legal US resident	3.7 (2.8–4.9)*	8.2 (5.2–13.3)*
Believe actions in anti-immigrant statements are being done or will be done	2.5 (1.8–3.6)*	2.3 (1.1–5.3)*
**Characteristic**	**aOR (95% CI)**	**aOR (95% CI)**
Age (unit = 10 years)	0.9 (0.8–1.0)	1.0 (0.9–1.2)
Female	2.1 (1.6–2.8)*	0.9 (0.6–1.4)
Identify as Latino	1.5 (1.1–2.1)*	1.2 (0.5–3.0)
Not legal US resident	2.8 (2.1–3.9)*	7.4 (4.4–13.3)*
Believe actions in anti-immigrant statements are being done or will be done	2.4 (1.6–3.5)*	2.1 (1.0–4.8)*

OR = odds ratio; aOR = adjusted odds ratio; CI = confidence interval; US = United States; ED = emergency department

*significant at p < .05

In terms of the secondary analyses of the perceived effects of these statements, UDLI living in the US for > 5 years had similar prevalence of safety concerns as UDLI who had recently immigrated (82% vs 74% [difference 8%, 95% CI -5 to 18%]) and similar rates of fear of coming to the ED (23% vs 32% [difference -10%, 95% CI -23 to 2%]). We found a higher rate of safety concerns in the last six months of sampling (July–December 2018) as compared to the first year (83.1% vs 72.8% [difference 10.3%, 95% CI 0 to 19%]), but similar rates of fear coming to the ED (23.3% vs 24.2% [difference -0.9%, 95% CI -10 to 10%]).

## Discussion

Excluded from the Affordability Care Act (ACA) and with limited options for regular health care, UDLI in the US report worse health and less health care utilization compared to US-born Latinos [17–19]. Given that the ED serves as the sole health care access point for many UDLI, efforts to improve UDLI health must begin with minimization of barriers (both true and perceived) to ED utilization. In this study we sought to assess whether statements about immigrants made during the 2016 presidential campaign or by President Trump have generated another barrier to UDLI access to EDs. We found that the vast majority of UDLI had heard statements about President Trump’s intended measures against undocumented immigrants and that most believed that these measures are being or will be enacted. According to responses in this survey, UDLIs reported that these statements have affected their feelings of safety living in the US and have induced fear of accessing the ED for health care.

Our UDLI population was not a medically naïve group—the vast majority had seen a doctor in the US previously and were aware that health care workers do not report patients to immigration authorities; few believed that doctors and nurses treat them differently. That nearly a quarter of them still had fear in coming to the ED attests to the potential power and threat of statements about deportation and denial of services coming from the president or being used during a presidential campaign. Notably, rates of safety concerns and fear of accessing the ED were similar in recent and non-recent immigrants, indicating a pervasiveness that does not appear to wane over time living in the US. The reasons for a higher prevalence of safety concerns in the latter part of the study are unclear, but it is possible that it arises from later statements and policy changes by the presidential administration, including narrowing asylum eligibility and changing the definition of “Public Charge,” such that immigration officials may consider a much broader range of public benefits when determining who is likely to become dependent on government benefits. These changes have the potential to lead to the disenrollment of millions of children from benefits that support their health and nutrition [20–22].

Beyond effects on the UDLI population, we have additionally demonstrated that the statements made by the president or during the campaign also correlate with perceptions held by LLRC, half of whom replied that these statements made them feel unsafe living in the US. Self-identification as Latino was independently associated with this feeling of being unsafe. These findings about safety are in line with reports that hate crimes against Latinos in California increased over 50% from 2016 to 2017 [23]. Although our NLRC enrollment group was intended to serve primarily as a control, a third of these patients replied that they had personal safety concerns arising from these statements about immigrants as well.

Undocumented immigrant populations in the US are a difficult group to study, and it is not surprising that little original research has been published regarding their barriers to accessing health care. Beyond the standard barriers of language difficulties and lack of financial resources, investigators have more recently reported that spikes in immigration enforcement were associated with decreased Medicaid enrollment [24,25]. This “chilling effect” on enrollment extends beyond UDLIs to their legal resident family members, as immigration raids have been linked to worse self-reported health and increased stress levels among legal residents of Latino communities [25,26].

Outside of efforts in the political arena, what can be done to reduce undocumented immigrants’ fear of accessing emergency care? The most obvious measure is to spread the message that US law guarantees everyone—regardless of citizenship—the right to emergency care. Many EDs promote *Know Your Rights* education initiatives with multiple language signs in waiting rooms and verbal reassurance at triage [27,28], but these messages may not reach those in greatest need of this information—immigrants who completely avoid coming to the hospital. Other measures to engage this hard-to-reach group include public awareness campaigns on Spanish media and forging community alliances through churches and neighborhood immigrant centers. Given that most UDLI knew that hospital staff do not report immigrants, merely spreading this message of safety in the ED may be insufficient. Addressing health disparities requires consideration of traditional social determinants of health (housing instability, food insecurity and limited English proficiency), as well as exploration of how other newly recognized factors, such as “racialized legal status,” may disproportionately affect their health [29–32].

Beyond the ED, tempering the rise in personal safety concerns in Latino populations may be even more difficult. A statement released by the Los Angeles Police Department in early 2018 showed substantial drops in the reporting of sexual assaults and domestic violence by the Hispanic community after the president’s inauguration [33]. According to a number of media reports, the mass shooting that targeted Latinos in El Paso, Texas, in August 2019 may have made UDLIs apprehensive about their personal safety and reluctant to report crimes because of their immigration status [34,35]. Similar to the message that everyone can safely receive care in the ED, initiatives assuring safety when seeking police assistance should also be considered. Our finding that LLRC and NLRC also had safety concerns rooted in the president’s statements about immigrants may signal the potential mental health benefit of such interventions for the broader US citizen population as well.

### Limitations

Although we optimized survey content with health literacy experts and pilot tested our survey instrument to ensure comprehension and response consistency, we did not specifically address other elements of instrument validity. Confidentiality constraints limited formal review of citizenship and use of self-reports for categorization of citizenship status may have led to misclassification of undocumented immigrants who may have been afraid to respond candidly. However, on review of ED registration records, we found similar rates of social security numbers in the LLRC and NLRC groups and low rates of social security numbers in UDLI, bolstering the face validity of this method of participant classification. Furthermore, self-report is the generally accepted standard for determination of citizenship in studies of this vulnerable population [26,32]. Our convenience sampling during weekday hours may have introduced spectrum bias.

Perhaps the greatest limitation of our work is that we surveyed patients in only one state and in communities that are generally considered to be *safe* for undocumented immigrants; UDLI in other states or communities may have higher or lower levels of fear. Similarly, because we only examined Latinos, our findings may not generalize to other immigrant populations. Another major limitation is that we only surveyed immigrants who actually came to the ED for health care—a quarter of UDLI and LLRC stated they had friends or family members who had not come to the ED because of fear, and we were unable to interview this group. These limitations all likely lead to an underestimation of the true levels of UDLI safety concerns and fear of accessing emergency care, and our study therefore likely provides lower-bound point estimates of what would be seen in a national sample of undocumented immigrants.

Finally, we must acknowledge that this topic is emotionally charged and that simply asking these questions may have paradoxically triggered fear in respondents. Social desirability may also have induced patients to answer in ways that they thought we wanted them to respond [36]. We attempted to mitigate these effects by training our interviewers to read the questions directly off the survey template in a standardized fashion with neutral tones and without insertion of other leading statements. Although we included IRB-mandated language in the scripted consent assuring patients that the study would not affect their care and that they would receive information about health care access, we avoided other language that would induce positive responses to our primary outcome questions. To diminish primacy bias (the tendency for respondents to choose the first option in surveys) toward finding greater effect, we placed neutral responses, e.g. “No, these statements have *not* affected me at all”, as the first choice in all of our queries. Our tiered decreasing rates of safety concerns from highest in UDLI to lowest in NLRC (as evident in Fig 1) logically support the validity of our central finding that the two Latino groups truly have heightened fear.

## Conclusions

The vast majority of patients in our California EDs have heard statements about deporting and denying services to undocumented immigrants from President Trump or during the 2016 presidential campaign. Latino patients (both UDLI and LLRC) responded that such statements made them feel unsafe living in the US. Some UDLI also indicated that these statements made them afraid to come to the ED and thus represent a potential psychological barrier to emergency care access.

## Supporting information

S1 Fig Survey English version. (DOCX)

S2 Fig Survey Spanish version. (DOCX)
