# Supplementary material for: Declared impact of the US President’s statements and campaign statements on Latino populations’ perceptions of safety and emergency care access
Source: PLoS One. 2019 Oct 30;14(10):e0222837. doi: 10.1371/journal.pone.0222837 (PMC6821049; doi:10.1371/journal.pone.0222837)
Supplement: S1 Fig — (DOCX) [file pone.0222837.s001.docx]

**A Follow-Up Study Of The Impact Of Fear Of Discovery On Latino Immigrants’ Presentations To The Emergency Department**

**Case Report Form**

*Ensure subject has been seen by an ED provider before approaching*

Data Collector Only

**E.** Is there a SSN listed on the EMR? Yes No

**F.** ESI level: ______

**G.** Study Group: Undocumented Latino Immigrant

 Legal Latino Resident/Citizen

- Non-Latino Legal Resident/Citizen
- Undocumented Non-Latino

**H**. Hospitalized? Yes No

*Ensure confidentiality*

**Verbal consent provided?:**

- **Yes** 🡪 **Proceed to survey**
- **No** 🡪 **Terminate survey**

**I. DEMOGRAPHICS:**

**A.** Age:____

**B.** Gender:
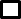
 Male
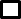
 Female
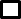
 Other:______

**C**: Do you *identify* as being of Latino origin?
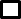
Yes
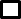
 No

**D.** Do you have a Social Security Number? Please ONLY answer **
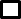
**Yes or
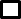
 No

**II. ALL SUBJECTS:**

**A.** What is your primary language?

- - English
  - Spanish
  - Cantonese/Mandarin
  - Armenian
  - Other: _______________________

**B.** Do *you* speak and understand English?

- - Not at all 🡪 B2
  - A little 🡪 B2
  - Most of it 🡪 C
  - All of it (completely) 🡪 C

**B2.** Does your *ED provider* (MD/NP/PA) speak the language you prefer (i.e. Spanish)?

- - - Not at all 🡪 B3
    - A little 🡪 B3
    - Most of it 🡪 C
    - All of it 🡪 C

**B3.** Was there an interpreter used?

- - - - None
      - Telephone / video
      - Another health care worker
      - Other: ______________

**C.** Do you have health insurance:
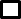
 Yes
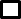
 No
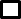
 I am currently applying for it
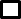
 Unsure

- If YES, what type:
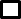
 Private
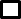
Medicare
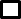
 MediCal
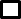
 Kaiser
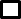
 Healthy SF/LA CARE
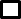
 ObamaCare
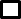
 Other

**D.** Do you currently have a place to live:
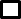
 Yes
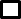
 No

- If YES, how long have you lived there?
  - - < 1 month
    - 1-6 months
    - 6 months – 1 year
    - > 1 year
- If NO, how long have you been homeless?
  - - < 1 month
    - 1-6 months
    - 6 months – 1 year
    - > 1 year

**E.** Do you have a regular clinic or doctor for medical care?
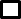
 Yes
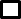
 No

- If YES, when was the last time you saw this doctor?
  - - < 1 month
    - 1-6 months
    - 6 months – 1 year
    - > 1 year
- If NO, when was the last time you saw any doctor in the U.S.?
  - - < 1 month
    - 1-6 months
    - 6 months – 1 year
    - > 1 year
    - I have never seen a doctor in the U.S.
- If NO regular doctor, where do you usually receive medical care?
  - - Clinic
    - An emergency department
    - Other________________________
    - I have never seen a doctor in the U.S.

**F. Are you a legal resident/citizen of the United States?
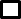
 Yes
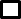
 No
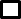
 Unsure**

If NO or UNSURE, were you brought to the United States illegally as a child?
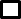
 **Yes
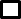
 No**

If YES, what age:_______

**G.** How long have you been in the United States?

- - - I was born here (all my life)
    - > 10 years
    - 5-10 years
    - 1-5 years
    - 6 months - 1 year
    - < 6 months
    - I don’t live here, I am just visiting

**H.** Do you believe that doctors and nurses treat US citizens/residents differently than non-US residents/citizens?
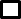
Yes
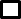
 No
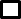
 Unsure

- If YES, how do they treat them differently?____________________________________________

**I.** Before this interview, did you believe that doctors and nurses report non-US residents/citizens to immigration authorities?
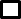
 Yes
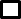
No
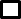
 Unsure

**J.** Before this interview did anyone in the hospital ask you whether you are a US citizen/resident?


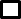
 Yes
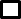
 No
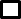
 Unsure

If YES, who?: ______________

**K.** On a scale of 1-10, how satisfied are you with the health care you received today?

(1 = not at all satisfied 10= very satisfied) ________

**L**. Do you know who the president of the U.S. is?
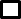
 Yes
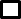
 No

If YES, who is it?_______________________________________________

**M**. Have you heard statements about immigrants during the presidential campaign or from President Trump?

- - Yes 🡪 go to M1
  - No 🡪 N then Terminate Survey

**M1**. If YES, what have you heard?

_________________________________________________________________________

**M2**. Have you heard any of these? Check all that apply.

- - President Trump wants to build a wall
  - President Trump wants to deport immigrants
  - President Trump wants to deny services to immigrants
  - President Trump wants to prevent immigrants from working here.
  - President Trump wants to prevent immigrants from getting health care in the U.S.
  - Other: _____________________________________________________________
  - No

** Keeping the list of M2 in mind (may repeat list for questions M3-M7)**

**M3**. Do you believe any of these things are being done now or will happen?

- No, not right now and never will happen 🡪 M5
- Not right now, but will happen in the future 🡪 M4
- Some are being done now 🡪 M4
- All are being done now 🡪 M4

**M4.** Which things do you think are being done now or will happen in the future?

____________________________________________________________________________

**M5.** Have these statements made you feel worried or unsafe living in the U.S.?

- No, these statements have *not* affected me at all.
- Yes, these statements have made a *little* worried or unsafe.
- Yes, these statements have made me feel *somewhat* worried or unsafe (a moderate amount).
- Yes, these statements have have affected me a *lot*. They have made me feel very worried or unsafe.

**M6**. Did any of these statements make you afraid to come to the emergency department?

- No, not at all
- Yes, a little 🡪 M7
- Yes, a moderate amount 🡪 M7
- Yes, a lot 🡪 M7

**M7.** If YES, did you delay the time it took for you to come to the ER today?

- - - - No
      - Yes, how long did you delay? ___________________ (# of hours, days, weeks, etc)

**N.** Do you know of someone (e.g., family or friend) that has not gone to the emergency department out of fear of being discovered as undocumented (non-U.S. resident/citizen)?
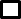
 Yes
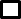
 No

**N1**. If YES, how many? _______________

**N2**. If YES, *who* told them that they should worry about this or *where* did they hear that they should worry about this? Mark all that apply.

- Friends or family
- Television or radio
- Other________________________
