# Supplementary material for: Declared impact of the US President’s statements and campaign statements on Latino populations’ perceptions of safety and emergency care access
Source: PLoS One. 2019 Oct 30;14(10):e0222837. doi: 10.1371/journal.pone.0222837 (PMC6821049; doi:10.1371/journal.pone.0222837)
Supplement: S2 Fig — (DOCX) [file pone.0222837.s002.docx]

**Un Estudio del Impacto del Miedo al Descubrimiento en las Presentaciones de los Inmigrantes Latinos al Departamento de Emergencias**

**Case Report Form**

*Ensure subject has been seen by an ED provider before approaching*

*Ensure confidentiality*

Data Collector Only

**E.** Is there a SSN listed on the EMR? Yes No

**F.** ESI level: ______

**G.** Study Group: Undocumented Latino Immigrant

 Legal Latino Resident/Citizen

- Non-Latino Legal Resident/Citizen
- Undocumented Non-Latino

**H**. Hospitalized? Yes No

**Verbal consent provided?:**

- **Yes** 🡪 **Proceed to survey**
- **No** 🡪 **Terminate survey**

**I. DEMOGRAPHICS:**

**A.** Edad:____

**B.** Sexo:

- Masculino Femenina Otro______

**C**: ¿Se identifica como de origen latino?? Si o No

**D.** ¿Tiene un Número de Seguro Social? Por favor responda SOLAMENTE Si o No

**II. ALL SUBJECTS:**

**A.** ¿Cual es su idioma principal?

- Ingles
- Español
- Cantonés / Mandarín
- Armenio
- Otro: _______________________

**B.** ¿Habla y entiende inglés?

- Nada 🡪 B2
- Un poco🡪 B2
- La mayor parte 🡪 C
- Todo (completamente) 🡪 C

**B2.** ¿Su *proveedor de servicios de emergencia* (MD / NP / PA) habla el idioma que prefiere (es decir, español)?

- - Nada 🡪 B3
  - Un poco 🡪 B3
  - La mayor parte 🡪C
  - Todo (completamente) 🡪 C

**B3.** ¿Se utilizó un intérprete?

- - - Ninguno
    - Teléfono / video
    - Otro trabajador(a) de la salud
    - Otro: ______________

**C.** Tiene seguro médico: Si No estoy solicitando
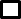
 No estoy seguro(a)

- Si? ¿qué tipo?
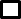
 Privado
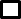
 Medicare
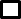
 MediCal
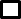
 Kaiser
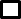
 Healthy SF/LA CARE
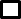
 ObamaCare
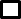
 Otro

**D.** ¿Tiene un lugar para vivir?
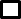
 Si
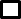
 No

- Si es SI, ¿cuánto tiempo ha vivido allí?
- < 1 mes
- 1-6 meses
- 6 meses– 1 año
- > 1 año
- Si NO, cuánto tiempo ha estado sin hogar?
  - - < 1 mes
    - 1-6 meses
    - 6 meses– 1 año
    - > 1 año

**E.** ¿Tiene una clínica o un médico regular para recibir atención médica?
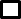
 Si
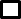
 No

- Si contestó SI, ¿cuándo fue la última vez que vio a este médico?
  - - < 1 mes
    - 1-6 meses
    - 6 meses– 1 año
    - > 1 año
- Si NO, ¿cuándo fue la última vez que utilizó algún médico en los Estados Unidos?
  - - < 1 mes
    - 1-6 meses
    - 6 meses– 1 año
    - > 1 año
    - Nunca he visto a un médico en los Estados Unidos.
- Si NO tiene un médico regular, ¿dónde recibe atención médica por lo general?
  - - Clinica
    - Un departamento de emergencias
    - Otro________________________
    - Nunca he visto a un médico en los Estados Unidos.

**F. ¿Es usted residente legal/ciudadano(a) de los Estados Unidos?
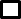
 Si
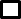
 No
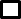
 No estoy seguro(a)**
Si NO o NO SEGURO(A), ¿fue traído(a) ilegalmente a los Estados Unidos cuando era niño(a)? **
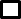
 Si
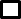
 No**

Si contestó SI, ¿qué edad?:_______

**G.** ¿Cuánto tiempo lleva en los Estados Unidos?

- Yo nací aquí (toda mi vida)
- 10 años
- 5-10 años
- 1-5 años
- 6 meses - 1 año
- < 6 meses
- No vivo aquí, solo estoy de visita

**H.** ¿Cree usted que los médicos y las enfermeras tratan a los ciudadanos/residentes legales de los Estados Unidos de manera diferente en comparación a los que no son ciudadanos/residentes legales?


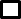
Si
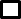
 No
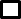
 No estoy seguro(a)

- Si es SI, ¿Cómo los tratan de manera diferente?__________________________________________

**I.** Antes de esta entrevista, ¿Creía usted que los médicos y las enfermeras reportan a las personas que no son ciudadanos/residentes legales de los Estados Unidos a las autoridades de inmigración?
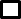
Si
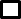
 No
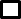
 No estoy seguro(a)

**J.** Antes de esta entrevista, ¿Alguien en el hospital le preguntó si usted es ciudadano/residente legal de los Estados Unidos?
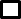
Si
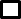
 No
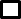
 No estoy seguro(a)

Si es SÍ, ¿quién?: ______________

**K.** En una escala de 1-10, ¿qué tan satisfecho(a) está con la atención médica que recibió hoy?

(1 = Nada satisfecho(a)10= Muy Satisfecho(a)) ________

**L.** ¿Sabe quién es el presidente de los Estados Unidos?
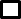
 Si
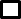
 No

Si es SI, ¿quién es?_______________________________________________

**M**. ¿Ha escuchado comentarios sobre inmigrantes durante la campaña presidencial o del presidente Trump?

- - Si 🡪 M1
  - No 🡪 N then Terminate Survey

**M1**. Si es SI, ¿qué ha escuchado?

_________________________________________________________________________

**M2.** ¿Ha escuchado alguno de estos? Marque todo lo que corresponda.

- - Presidente Trump quiere construir un muro
  - Presidente Trump quiere deportar inmigrantes
  - Presidente Trump quiere negar servicios a inmigrantes
  - El Presidente Trump quiere evitar que los inmigrantes trabajen aquí
  - El presidente Trump quiere evitar que los inmigrantes obtengan atención médica en los Estados Unidos.
  - Otro: _____________________________________________________________
  - No

** Keeping the list of M2 in mind (may repeat list for questions M3-M7)**

**M3.** ¿Cree usted que alguna de estas cosas se está pasando ahora o va a suceder?

- No, no en este momento y nunca sucederá 🡪M5
- No en este momento, pero sucederá en el futuro🡪 M4
- Algunos se están pasando ahora🡪 M4
- Todo se está pasando ahora🡪 M4

**M4.** ¿Qué cosas cree que se están pasando ahora o van a suceder en el futuro?

____________________________________________________________________________

**M5.** ¿De estos comentarios, le han hecho sentir preocupado o inseguro de vivir en los Estados Unidos?

- No, estos comentarios *no* me han afectado en absoluto
- Sí, estos comentarios han hecho *un poquito* preocupado o inseguro
- Sí, estos comentarios me han hecho sentir *un poco* preocupado o inseguro (una cantidad moderada)
- Sí, estas comentarios me han afectado *mucho*. Me han hecho sentir muy preocupado o inseguro

**M6.** ¿Alguno de estos comentarios le hizo temer ir al departamento de emergencias?

- No, en absoluto
- Sí, un poco 🡪 M7
- Sí, una cantidad moderada 🡪 M7
- Sí, mucho 🡪 M7

**M7.** Si es sí, ¿retrasó el tiempo que le llevó a venir a la sala de emergencias hoy?

- - - - No
      - Sí, ¿cuánto tiempo se tardo?___________________ (# horas, dias, semanas, etc)

**N**. ¿Conoce a alguien (por ejemplo, familia o amigo) que no ha ido al departamento de emergencia por temor de ser descubierto como indocumentado (no residente/ciudadano de los Estados Unidos)?
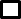
 Si
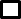
 No

**N1**. Si es SI, ¿cuántos?_____________

**N2.** Si es SI, ¿*Quién* les dijo que deberían preocuparse por esto o *dónde* se enteraron de que deberían preocuparse por esto? Marque todo lo que corresponda.

- Amigos o familia
- Televisión o radio
- Otro________________________
